# Building value for dairy farmers and advisors in the Farmers Assuring Responsible Management Environmental Stewardship Program

**DOI:** 10.1093/tas/txaf038

**Published:** 2025-03-25

**Authors:** MaryGrace Erickson, Maristela Rovai, Patricia Villamediana, Amy M Schmidt, Richard R Stowell, Erin L Cortus

**Affiliations:** Department of Bioproducts & Biosystems Engineering, University of Minnesota, St. Paul, MN 55108, USA; Department of Dairy and Food Science, South Dakota State University, Brookings, SD 57007, USA; Department of Dairy and Food Science, South Dakota State University, Brookings, SD 57007, USA; Department of Biological Systems Engineering, University of Nebraska-Lincoln, Lincoln, NE 68583, USA; Department of Animal Science, University of Nebraska-Lincoln, Lincoln, NE 68503, USA; Department of Biological Systems Engineering, University of Nebraska-Lincoln, Lincoln, NE 68583, USA; Department of Animal Science, University of Nebraska-Lincoln, Lincoln, NE 68503, USA; Department of Bioproducts & Biosystems Engineering, University of Minnesota, St. Paul, MN 55108, USA

**Keywords:** assessment, environment, qualitative, perceptions, sustainability

## Abstract

Major industry-led efforts aim at reducing the cradle-to-farmgate environmental impacts of milk production (e.g., U.S. Dairy Net Zero Initiative). Our qualitative, exploratory work sought to characterize farmer and advisor perceptions of an environmental sustainability assessment program [FARM ES Version 2] in the Upper Midwest. We aimed to 1) explore the ways participants valued environmental stewardship (ES) assessments, 2) describe feasible on-farm improvement strategies identified regionally, and 3) characterize how participants assigned responsibility for enacting selected improvement strategies. In Fall 2023, we held a series of 2 to 3 focus groups for each of 5 regions (n = 14 meetings total) including farmers (n = 24), advisors (n = 20), and processor representatives (n = 1). Facilitators guided participants through semi-structured prompts to generate qualitative data including meeting transcriptions, consensus lists, and consensus diagrams. First, we used a deductive-inductive process to analyze meeting transcriptions and identify themes related to the value of environmental stewardship assessments. Results suggested that participants valued simplicity, ease of data entry, availability of regional comparisons, and the ability to enumerate a baseline for environmental performance. Conversely, participants reported skepticism about accuracy and fairness and the usefulness of assessments as decision-support tools. Second, we examined consensus documents to generate a list of feasible strategies for on-farm improvement. Participants identified immediately feasible management strategies including cover cropping, genetic improvement, no-/minimum-tillage, precision nutrient management, herd/facility management technologies, monensin supplementation, and the conversion of fossil fuel to electric motors. Finally, we inspected collaborative actor-network diagrams generated with participants, which illustrated that participants envisioned implementation as complex and (in some instances) contingent on cooperation across supply chains and allied industries. Overall, our findings suggested that dairy farms need both accessible entry points into ES management and advanced technical and social support for implementing changes.

## Teaser

This study revealed how farmers and advisors in the U.S. Upper Midwest interpreted the Farmers Assuring Responsible Management Environmental Stewardship Program.

## INTRODUCTION

In 2020, the U.S. Dairy organization launched the Net Zero Initiative, an industry-wide effort to promote voluntary efforts to reduce environmental impacts associated with milk production (U.S. Dairy, 2023). The Net Zero Initiative set 3 goals for the dairy industry: 1) achieving greenhouse gas neutrality, 2) optimizing water use while maximizing recycling, and 3) improving water quality (U.S. Dairy, 2023). In addition to supporting research and market development, the Net Zero Initiative has accelerated the implementation of environmental stewardship assessments. For example, in 2017, the dairy industry’s leading measurement and verification program (Farmers Assuring Responsible Management [**FARM**]) launched an environmental stewardship area. The FARM environmental stewardship (**FARM ES**) program provides a parsimonious assessment for estimating the cradle-to-farmgate greenhouse gas footprint of dairy farms based on a national life cycle assessment (Asselin-Balençon et al., 2013), along with an inventory of conservation practices. In addition to engaging producers in ES improvement efforts, the FARM ES program enables aggregation and reporting of environmental impacts across the dairy supply chain including for producers and retailers (FARM ES, 2023). Currently, participation in the FARM ES program is voluntary. The second-party assessment is most often facilitated by trained representatives of milk processors. Because the FARM ES program offers an industry-accepted, standardized method for estimating and reporting dairy environmental impacts, it may become a primary means for farms to assess and improve farm environmental performance.

Although many individual producers collect and monitor farm environmental data individually, coordinating environmental assessments across the dairy industry is unprecedented. Limited research has assessed how dairy farmers, processors, and members of allied industries perceive industry-wide ES efforts. We structured focus group discussions to address three research questions (**RQ**): RQ1: In what ways do participants value environmental stewardship assessments using the FARM ES program as an example? RQ2: Which on-farm improvement strategies do participants identify as accessible and profitable for their region? RQ3: Where (or to whom) do participants assign responsibility for enacting each improvement strategy? Therefore, in the current research, we conducted a qualitative inquiry into stakeholder perceptions of dairy ES assessment and improvement in five dairy-intensive regions in the Upper Midwest.

## METHODS

The University of Minnesota Institutional Review Board reviewed all study procedures. We conducted a qualitative, exploratory inquiry through a series of focus groups with dairy farmers and advisors in 5 regions in the Upper Midwest. Qualitative, inductive designs are well-suited for research in emerging topic areas where key variables and theory are not yet defined (Creswell, 2002). We adopted a pragmatic approach, using the dominant environmental assessment program in the U.S. dairy industry (FARM ES) as a foundation for focus group discussions and consensus-building on environmental stewardship assessments more generally (Morgan, 2014). Participants discussed revisions and additions to the FARM ES Version 2 reports and resources. Consequently, our work has immediate relevance to many U.S. dairy producers, although our conceptualization of environmental issues and assessments is focused narrowly on issues currently addressed by the U.S. Dairy Net Zero Initiative and the FARM ES program.

The authors note the FARM ES program is evolving. FARM ES Version 3 platform was released in October of 2024 with substantial updates to the calculation methods. There were only small changes to the format and content of the ES reports—the product of interest of this study. Thus, the results of this study should be transferable to Version 3.

### Participant Recruitment and Characteristics

Our study aimed to characterize the implementation of environmental stewardship assessments among the dairy production systems of 3 major dairy states in the Upper Midwest: Minnesota, Nebraska, and South Dakota. Across the three-state region, diverse environmental, social, and economic conditions support dairy production and environmental stewardship. To gain insight into the total system of dairy and supporting industries involved in environmental assessments, we employed a two-stage purposeful sampling strategy (Creswell, 2002). First, based on the study team’s prior experience, we selected 5 locations within the three-state area as regional centers for focus group activities: Central Minnesota (MN1), Southeast Minnesota (MN2), East-central South Dakota (SD1), Southeast South Dakota (SD2), and Northeast Nebraska (NE1). Each regional center exemplified distinct socioenvironmental characteristics (i.e., average herd size, environmental pressures) and served as an intermediate unit of analysis and a starting point for recruiting participants (Yin, 1994).

For each region, study team members appointed as extension professionals in that region led the recruitment and communication processes with participants. We selected participants purposefully to maximize the variety of farmer and advisor characteristics represented within close geographic proximity to the selected region. Using personal contacts and public contact information for companies online, the study team approached potential participants via phone and email representing the following roles: dairy farmer, nutritionist, veterinarian, veterinary services, Natural Resources Conservation Service, electric provider, farmer organization representatives, feed industry representatives, processor representatives, and agricultural equipment dealers. To be included in the study, participants needed to have had recent (within the last 5 yr) experience working directly with dairy farms. By engaging a diverse group of participants from each of these distinct regions, we aimed to represent a broad cross-section of the dairy industry in the U.S. Upper Midwest. Our qualitative approach aimed at transferable and trustworthy results rather than statistical representativeness and generalizability (Ritter et al., 2023).

### Focus Group Design and Facilitation

Throughout Fall 2023, we held three focus group events in each of four regions, designated MN1, MN2, SD1, and SD2. In the fifth region, NE1, two focus group events were held due to scheduling constraints, with the second event being lengthened so the same research questions were covered as in the other regions. Discussion at each focus group event lasted 1.5 to 3 h. The focus group discussions were led by study team members appointed as extension professionals in that region, who had professional relationships with participants. This familiarity between facilitators and participants may promote honest information-sharing, enhancing the credibility of findings (Shenton, 2004).

We limited discussions to small groups of 5 to 10 participants to enhance the representation of individual voices (Onwuegbuzie and Leech, 2007). Within each region, most participants attended 2 to 3 meetings. In total, unique participants represented a similar number of farmers and advisors (n = 24 and n = 20, respectively; Table 1). Participants represented farm sizes ranging from approximately 120 to 11,000 lactating cows. The focus group sample size, duration, and sequence design were guided by recommendations for achieving thematic saturation (Hennink and Kaiser, 2022). Participants were not compensated but received a complimentary meal before or during the discussion.

**Table 1.** Count of participants in focus groups for five regions categorized by their role in the dairy industry

Region^1^	Farmer	Advisor	Processor	Total
Event 1				
MN1	1	3	1	5
MN2	2	5	0	7
SD1	8	2	0	10
SD2	8	2	0	10
NE1	3	1	0	4
Event 2				
MN1	2	3	0	5
MN2	2	4	0	6
SD1	8	2	0	10
SD2	5	2	0	7
NE1	4	3	0	7
Event 3				
MN1	1	4	0	5
MN2	0	2	0	2
SD1	9	2	0	11
SD2	4	2	0	6
NE1^2^	N/A	N/A	N/A	N/A
Total Unique	24	20	1	45

^1^.Regions included Central Minnesota (MN1), Southeast Minnesota (MN2), East-central South Dakota (SD1), Southeast South Dakota (SD2), and Northeast Nebraska (NE1).

^2^.For NE1, event 2 and 3 meeting contents were combined due to time constraints. N/A indicates “not applicable.”.

Facilitators used a series of prompts to guide a semi-structured discussion, as summarized in Table 2. Each focus group event for a region focused on one RQ, except the second focus group event in NE1 that covered RQ2 and RQ3. These prompts were collected in a written facilitator guide to standardize discussion processes across the study team (Supplements 1–3). Each event had one to three facilitators. One facilitator (M.E.) was present for 13 of 14 focus group events, providing additional consistency in delivery and interpretation. For RQ1, facilitators distributed FARM ES Version 2 reports representing fictional farms—one small (150 lactating cows) and one large (1,500 lactating cows)—exemplifying typical production characteristics for the region. We created realistic region-specific input data for fictional scenarios based on state-average production data (USDA NASS, 2023), published reports on dairy operating characteristics, and personal communications with extension professionals in each region. After sharing initial impressions, facilitators guided a semi-structured discussion focused on the value of environmental assessments and potential improvements. For RQ2, to provide participants some common understanding and terminology, we invited them to revise and add to a list of science-based improvement strategies suggested in Net Zero Initiative documentation (U.S. Dairy, 2021; Supplement 4). Next, participants selected improvement strategies that were priorities in their region. Finally, facilitators guided participants through categorizing the perceived accessibility and profitability of these selected improvement strategies using a quadrant diagram (unprofitable [-] to profitable [+]; inaccessible [-] to accessible [+]). Improvement strategies rated as both accessible and profitable (or at least not resulting in financial losses) were considered feasible. For RQ3, facilitators presented a series of 2 to 3 improvement strategies and invited participants to comment on the actors, actions, and outcomes involved in implementation. Building on the work of Comi (2020), we conceptualized actors as both human (e.g., a dairy farmer) and non-human agents (e.g., a company or organization). During all activities, facilitators recorded impressions from participants on a large paper easel pad visible to the entire group to work toward a consensus opinion.

**Table 2.** Summary of activities in the sequence of focus group events pertaining to each research question (RQ)^1^

Prompts and Activities	Format	Data Outputs
RQ1		
A) reactions and interpretations of FARM ES inputs and outputs, B) the value in FARM ES report(s) for processors, farmers, advisors, and others	Semi-structured discussion Comparison of fictional reports	Meeting transcriptions^*^ Consensus list of valued aspects of assessment process Consensus list of revisions to fictional reports
RQ2		
A) management strategies that are priorities for implementation and further development for this region, B) accessibility and profitability of management strategies, C) exciting opportunities that may not yet be feasible	Semi-structured discussion Management ideation activity Quadrant activity	Meeting transcriptions Consensus list of management strategies for each category, for this region^*^ Consensus ratings of accessibility and profitability for strategies, for this region^*^
RQ3		
A) for this region, who and what can support improvement and implementation for this management strategy, B) What are the main outcomes of implementing this strategy	Semi-structured discussion Actor identification activity Actor-network mapping activity	Meeting transcriptions Consensus list of key actors involved in implementing the strategy, for this region Consensus network of actors, actions, and outcomes for this strategy and region^*^

^*^indicates a primary output used for analysis and reporting of results for the RQ.

^1^. RQ1: In what ways do participants value environmental stewardship assessments using the FARM ES program as an example? RQ2: Which on-farm improvement strategies do participants identify as accessible and profitable for their region? RQ3: Where (or to whom) do participants assign responsibility for enacting each improvement strategy?

### Qualitative Analysis

Our facilitation procedures produced two types of qualitative data 1) meeting transcriptions, which required post-discussion qualitative analysis to summarize and aggregate across regions, and 2) consensus items that required minimal post-discussion qualitative analysis and were aggregated by summing across regions. Initial qualitative analysis within region was led by each region’s focus group facilitators, as greater familiarity with participants and local context theoretically enhances the trustworthiness of qualitative findings (Shenton, 2004). Nonetheless, we held weekly debriefings with the multi-regional study team during and after the focus group series to enable peer scrutiny of interpretations. Later stages of qualitative analysis and aggregation across regions involved all members of the study team.

For RQ1, each focus group’s facilitators conducted an initial round of provisional coding using meeting transcriptions (Byrne, 2022). In this initial round, facilitators assigned deductive codes aligned with prompts in the facilitator guide (Supplement 1) and inductive codes describing how and why the assessment was valued (Williams and Moser, 2019). Then, a study team member (E.C.) aggregated across regions by mapping provisional codes onto axes to identify themes and superordinate categories similar to Wilson et al. (2021). Finally, the study team discussed themes and categories to resolve disputes and reach a consensus.

For RQ2 and RQ3, we primarily used consensus outputs from focus groups. Consensus lists and diagrams are methods of rapid qualitative approaches (Vindrola-Padros and Johnson, 2020) which involve participants in deriving major themes. When describing complex topics, working with participants to generate shared graphical depictions has the potential to produce more detailed descriptions of phenomena compared with analyzing meeting transcriptions. In contrast with post-discussion analysis of meeting transcriptions, consensus lists and diagrams enable participants and researchers to work together to derive and member-check findings during a focus group discussion (Wheeldon and Faubert, 2009). To summarize consensus lists across regions for RQ2, we tallied the number of regions rating an improvement strategy as accessible and profitable. To summarize consensus diagrams across regions for RQ3, we constructed actor-network diagrams that graphically represent the collection of actors, actions, and nondirectional relationships described by participants (simplified from Alexander and Silvis, 2014; Payne, 2017). When necessary, we referenced meeting transcriptions to clarify and elaborate on listed improvement strategies and actor-network relations.

### Background on the Upper Midwest Dairy Industry

Rohleder and Lyons (2017, p. 57) emphasized that an in-depth description of the study context can improve the transferability of results to new settings. Collectively, Minnesota, Nebraska, and South Dakota accounted for 7.3 billion kg (16.1 billion lbs) of milk production annually (7.1% of U.S. total) with 692,000 milk cows in 2,290 herds (USDA NASS, 2022). We selected Central and Southeast Minnesota as regional centers because these areas include large fractions of the state’s dairy farms (8.1% and 20.9% respectively, USDA NASS, 2017) and account for large fractions of the state’s milk cow population (7.6% and 22.9%, respectively, USDA NASS, 2023). We selected a regional center in Northeast NE because dairy producers and processors are more geographically concentrated in that portion of the state. A Nebraska Department of Agriculture report (2014) estimated that over half of the state’s milk production was shipped out of state for processing, primarily to northwestern Iowa. Finally, we selected regional centers in East-Central and Southeast South Dakota because the largest-producing counties and processors are located along South Dakota’s eastern border. Milk production and lactating cow populations were relatively consistent in Minnesota and Nebraska in the last decade. By contrast, South Dakota ranked among the fastest-growing dairy states in terms of milk production. From 2017 to 2022, the state’s yearly milk production grew from 1.27 to 1.90 billion kg (2.8 to 4.2 billion lbs, USDA NASS, 2023), largely driven by expanded processing capacity.

### Positionality

In qualitative research, the researcher is the main instrument for data collection and interpretation (Creswell, 2002). Positionality describes how a researcher or study team is situated within a context in ways that influence both how the researcher(s) see and interpret phenomena, and how the researcher(s) are perceived by participants (Jacobson and Mustafa, 2019). Our study team included 5 faculty members and 1 postdoctoral associate, who held appointments at Land Grant universities in research, teaching, and extension related to livestock environmental management. Most study team members (A.S., R.S., M.R., P.V., and E.C.) have held longstanding involvement as extension professionals within their state and, therefore, possess deep knowledge of local agricultural practices and familiarity with some study participants. However, our study team varied in the extent of prior involvement with the dairy industry. Some members held dairy-focused research and extension roles, whereas others worked across livestock species. Collectively, our study team primarily had experience with conventional livestock production systems. The prior knowledge and positioning of our team shaped the selection of regions, inclusion criteria for participants, and interpretation of results (Day, 2012).

## RESULTS AND DISCUSSION

Our study examined farmer and advisor perspectives on the implementation of environmental sustainability assessments in the Upper Midwest. We aimed to explore 1) valued aspects of the FARM ES Version 2 program, 2) on-farm improvement strategies identified as accessible and profitable for the Upper Midwest, and 3) actors and actions participants identified as responsible for ES.

### Value of Environmental Assessments

**Figure 1.**
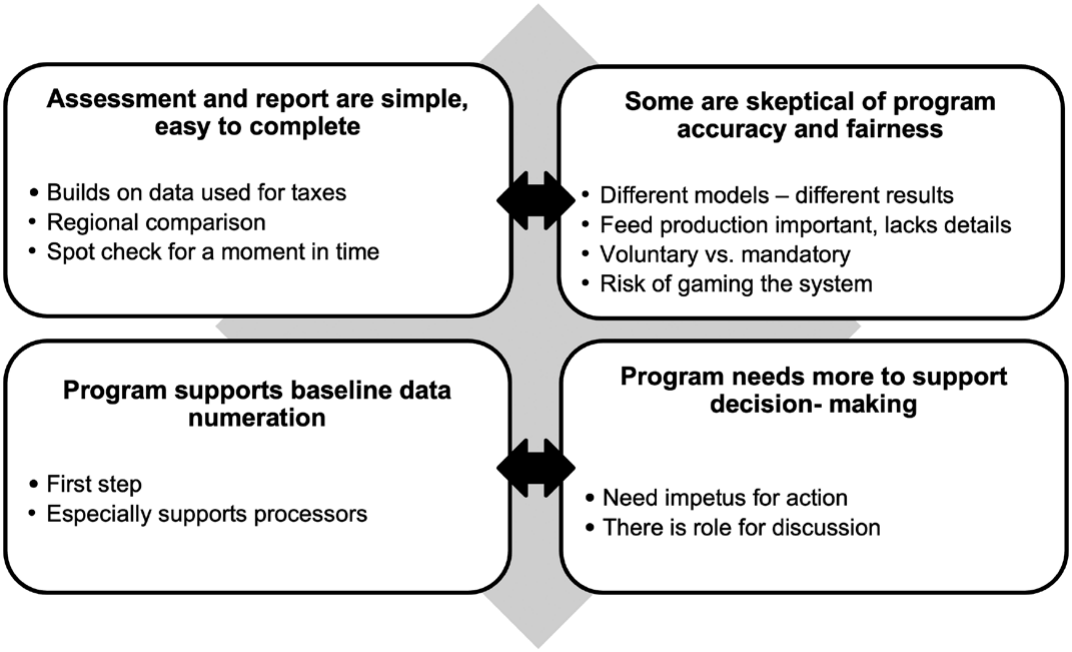
Categories and themes identified by farmer and advisor participants in focus groups in the U.S. Upper Midwest related to the value of the FARM ES Version 2 assessment.

Major categories and themes identified from farmer and advisor discussions are shown in Figure 1. We identified 2 overarching categories of themes. Each theme represented a trade-off associated with the assessment process. First, participants reported that the assessment process was a simple means to set a baseline or obtain regional benchmarks. However, certain participants commented that the assessment’s simplicity could contribute to inaccuracy and unfairness. Second, participants shared the FARM ES program was an accessible first step into environmental assessments. Furthermore, they reported that farmers and advisors would require additional support to make decisions and implement actions following the environmental assessment. Overall, our findings emphasize that farmers and advisors are discerning and action-oriented users of environmental assessment data.

#### Simplicity and fairness of assessment processes and accuracy of model results.

Farmer and advisor participants mentioned valuing the simplicity of the FARM ES assessment and report. When considering the assessment process, participants expressed that the time and effort required to collect input data was minimal. The FARM ES Version 2 requires participants to self-report the milk and component production, herd demographics, cull animal sales, lactating cow diet, dry matter intake, energy use from various sources, and manure management system(s) associated with a farm. These input data requirements did not appear to deter participation for any of the farms represented by participants in our research.

- *…it does take a little time, but you gather all that information for taxes for the most part so it’s there [MN1]*
- *Simplicity. This is easy to fill out. This isn’t awful [SD2]*

Importantly, the FARM ES process is typically mediated through a second-party evaluator, which decreases the technical burden on farmers and advisors in gathering and entering data. At the time of our research, data inputs had to be entered manually (i.e., there was no system automatically linking farm information systems to the FARM ES assessment). New data platforms have emerged to address administrative demands on farmers, who may participate in multiple private and public environmental performance schemes (Reissig et al., 2022).

It was evident from the focus groups that participants in our study valued both simplicity and accuracy and that they viewed these factors at times in opposition.

- *But I think maybe it’s a balance too between what makes it simple to gather the data and then also what’s going to produce accurate estimates, too. [SD2]*
- *Well I’m sure it’s a balance, right, between accuracy and complexity of entering data. It can be so complex that nobody can do it and get it accurate. So, I’m sure that’s why they... I’m sure it’s just that balance. [MN1]*

Thus far, many environmental assessments (including FARM ES Version 2) utilize yearly inventory data. The collection of yearly data aligns with traditional federal agricultural statistics reporting (e.g., USDA NASS) and farm recordkeeping for tax purposes. However, using year-long averages may obscure seasonal dynamics with implications on environmental outputs, as demonstrated by process-based models (e.g., Rotz et al., 2022). Similarly, several participants mentioned dissatisfaction with the spatial resolution of inputs and the level of detail in representing cropping practices. One participant contrasted the simplified, aggregate representation of cropping practices in the FARM ES assessment of feed production impacts with their use of a detailed, field-level environmental assessment:

- *… A third of their footprint is the growing of the feed because you don’t even know anything about any of the practices here. And you use a fuel print calculator to go through each individual field. You’re doing a day’s worth the work in the first place. [MN2]*

Similarly, participants expressed a need for the assessment to reflect herd-to-herd and animal-to-animal differences in environmental performance. In particular, participants expressed doubts that improvements in animal health, longevity, and genetics would be reflected in environmental assessments:

- *Every animal has a footprint to it. [SD2]*
- *Unless I’m mistaken, isn’t there a huge spread even as far as the methane produced by an individual cow between the high- and low-end emitters? [NE1]*

Additionally, several participants commented that the environmental assessment was focused too narrowly on climate impacts and lacked information about other types of environmental impacts.

- *It does say environmental stewardship. It would be interesting if they did add in even just a risk of water contamination…. And obviously maybe this doesn’t have even enough information in there of how the manure is applied, that kind of thing to actually put that in the report, but if maybe you’d have to gather more information to be able to do that, but somehow… [MN1]*
- *I know a lot of the focus is on carbon, but it’s called the environmental stewardship. So what other things are farms doing? Not that I’d recommend it, but switch back to …or other environmental impacts that aren’t just a strict carbon footprint practice. [SD2]*

In particular, participants discussed how the assessment should address local nutrient and energy efficiency and circularity:

- *When you talk about feed efficiency, I would say the biggest room for improvement, probably, [is] when it comes to diet and putting up silage piles properly. [SD2]*
- *So how do you compare a farm that produces its own feed? It says a hundred percent, but we have dairies in our area that buy a hundred percent of their silage…that should be somewhere reflecting in the numbers. [SD1]*
- *So I’m not raising that corn on the farm. Is that corn coming from my neighbor across the road or is that corn coming from seven states away? I mean, that’ll affect the environmental footprint of that. [MN1]*
- *…the manure that comes from these farms…they reprocess it and use it for bedding, but there’s a value to that…it adds so much to the value of your future crops, and that type of thing. So you’re adding value to your soil by using that manure, so that’s like a basket full of goodies all in one, but I don’t see that it’s anywhere in here that’s added in. [MN2]*

Collectively, these comments suggest a desire among farmers to expand the environmental impacts represented in environmental stewardship assessments, moving away from focusing on carbon footprints and toward a holistic, indicator-based sustainability assessment.

Our focus group discussions suggested that participants judged the accuracy of an environmental assessment based on how adequately it represented local farm characteristics they perceived as important to environmental stewardship. This illustrates a tension in the spatial and temporal scales at which environmental impacts are conceptualized and felt throughout the dairy value chain. When considering regional or national scales, environmental assessments such as FARM ES Version 2 have been demonstrated to produce accurate aggregate estimates of environmental impacts while requiring minimal inputs and excluding certain inconsequential processes (Asselin-Balençon et al., 2013). However, farmers and advisors may judge the validity and usefulness of the assessment in relation to particular farms at the local scale. Indeed, farmer knowledge of environmental management may be constructed largely with regard to their own farming system (Nguyen et al., 2019; Griffin et al., 2023). As discussed by Forney and Epiney (2022, p. 179), overly rigid agri-environmental standards can conflict with “the irreducible specificity of farming operations and situations” and the influence of uncontrollable factors such as weather conditions. Several participants suggested that they were uncertain how well the assessment captured these fringe situations and region-specific details:

- *I don’t know how much this average is going to be accurate. In a simplistic way, I think it works, but I would like to know better how these calculations are made. Because there are big variation between clouds, between regions, between grids, between management, and all of that. [SD2]*
- *Southeast Minnesota is extremely different than North-Central Minnesota. And we’re in Minnesota. We’re all Minnesota. But those two regions are completely different from each other on, for example, milk production, milk quality, but also type of forages that are grown, quality of forages, and all that. So could be cultural as well. I think there’s a cultural switch between southeast Minnesota and central, right? [MN2]*

For many environmental assessments, including FARM ES Version 2, the calculations used to estimate environmental outputs are hidden from users. Some authors speculated that opaque algorithms can lead to a false sense of precision and accuracy that undermines checks and balances against the ground truth in agriculture (Visser et al., 2021). Our focus groups indicated a need for checking the model outputs against measured environmental outputs and impacts:

- *So we’re asking a lot of questions. Where do these numbers come from? What does this number mean? To your point, how does it compare to whatever? Now we opened the Pandora’s box. So this is what our numbers are, now we have to explain it. [NE1]*
- *I just hate that we’re assuming accuracy here…Is there a verifiability, something like that? “How do you know a number is real and accurate?” is what we’re getting at. [NE1]*
- *How do they measure? Do they measure air? Do they measure dirt? Nobody really knows. Everybody’s got that carbon footprint or carbon reduction. How do we know that number is right as far as carbon? [SD1]*

Several participants mentioned that environmental assessments had the potential to create inequities and incentivize poor behavior.

- *…these companies are just falling all over trying to figure out how they accomplish this. How do they accomplish this and validate it. And I’ve asked, we’ve been asked about things, so how are you validating it? It’s a land of mush. Nobody really knows. So I’m not afraid of it. But who’s keeping score and are we keeping score in a fair way to everybody…[SD1]*
- *So if they say, “We’ll you pay you $50 for every hundred pounds of milk if you do this, this and that,” and that’s going to be wrong because... And I think that’s what these numbers are pushing us towards, that they’re going to be able to incentivize us to get those numbers. Everybody would be lying like you do with taxes. [SD1]*
- *…are these numbers that are old? Are they wrong? Have they been tweaked on by somebody, maybe for self-interest, maybe not? All those are possibilities. [NE1]*

In principle, multi-stakeholder sustainability initiatives such as the FARM ES program are grounded in the democratic involvement of multiple stakeholder voices (Ponte, 2014). However, the degree of stakeholder involvement in developing, refining, and continued use of assessments can vary dramatically (Binder et al., 2010). The FARM ES assessment discussed in our research exemplifies a voluntary, metrics-based approach. Whereas standards-based approaches include cut-points or specify desirable levels of performance, metrics are a form of “soft governance” that allows for plural interpretations of results (Konefal, 2015). The inherent pluralism of metrics-based approaches may enable greater equity and inclusivity because farms are free to determine the extent and direction of their environmental stewardship actions (Ponte, 2014; Rosin et al., 2017). However, environmental stewardship assessments have the potential to be leveraged in power dynamics among actors. Rather than conforming to metrics schemes, farmers may attempt to rework the evaluative system or seek alternative metrics that cast them in a more favorable light (Pollock et al., 2018). Several authors pointed out how measures can be misused when actors “play to the test” by focusing on improving the metrics themselves rather than the underlying phenomena that the metrics measure (Espeland and Sauder, 2007; Slager et al., 2021). Our results suggested that farmers and advisors valued environmental assessments that promoted the equitable, inclusive participation of a wide variety of dairy farms.

#### Supporting initial and continued engagement with environmental assessments.

Clearly, the extent which farmers engage in environmental assessment processes depends on the value they perceive may be derived toward farm objectives. As one participant summarized:

- *Or to me, I’m a farmer. I go, “Okay, this is great. Who’s this for? What can I do with it?” I think that, to me, it would be “what’s next?” Make it valuable for the farmer, and they’ll want to do it and they’ll be more interested in it. [MN1]*

Participants voiced that the assessment provided a starting point for discussion and that model outputs could set a baseline for comparing a farm’s environmental performance over time.

- *…I think it might be a very good beginner step just for the farm to get them ruminating on things. [MN2]*
- *I think this gives the opportunity to have a comparison over time too. This report doesn’t have yet a graphic showing how you did the previous year, but the data here will give you the opportunity at least to see how things are progressing or regressing. [SD2]*

In addition to comparisons against a baseline, farmers and advisors conveyed an appreciation for the inclusion of comparisons against regional averages. Participants mentioned that including model inputs provided an important context for interpreting results.

- *…at least you’re able to compare to your peers in the area, so you get that on the printout. [MN1]*
- *Benchmarks are helpful to see where you’re lying in there. [SD2]*
- *So I like the point that it’s obviously a focus on the results pages, but from the dairy farm perspective, those inputs can be... just knowing what those inputs are can be just as valuable. [MN2]*

Some participants remarked that the FARM ES Version 2 assessment had limited value for farmers and instead primarily addressed needs for firms downstream in the supply chain.

- *I think it’s useful for National Milk and it’s useful for [Dairy Management Inc.], and it may be even useful to some degree then too for as the aggregators and for the processors, but very little of help for a farmer. [MN1]*
- *So some of my negative bias…Where do you come up with this stuff? Who makes these rules? So I’m a little nervous that there’s some of this behind the curtain here. [NE1]*

Many participants had prior experience with the FARM Animal Care Program, a standards-based program administrated by the National Milk Producers Federation. Through a mixed-methods survey, Rink et al. (2019) showed that dairy farmers generally had positive experiences with the FARM Animal Care Program evaluations, yet many farmers expressed a need for greater inclusion of producers in program design and administration. Indeed, research has shown that closer engagement between farms and metrics providers may promote action on sustainability metrics (Slager et al., 2021). Narratives surrounding voluntary environmental stewardship programs often describe benefits to farmers without explicitly mentioning benefits to others in the dairy value chain such as processors and retailers (Forney and Epiney, 2022). Technological advances have increased the volume and availability of data generated by dairy farms. Private firms increasingly collect and generate revenue from farm data--without necessarily sharing profits with farmers (Fraser, 2019). Currently, there are few laws regulating data usage and sharing in the dairy industry (Cue et al., 2021). Our findings suggest that farmers and advisors were generally open to sharing data for the purpose of environmental assessments. However, participants were critical of uncertain benefits to farmers in light of clearer benefits to processors and retailers. This illustrates that environmental assessment processes involve balancing costs and benefits to multiple stakeholders within the dairy industry.

The FARM ES Version 2 provided comparisons against static values for the regional average environmental performance of dairies in an industry-wide lifecycle assessment (Thoma et al., 2013). However, the FARM ES platform provides a data infrastructure to pool farm data at various scales (Kitchin and Lauriault, 2015). Thus, there is potential for dynamic benchmarking, in reference to other farms with similar operational characteristics, in similar climactic conditions, or in similar regulatory environments. Participants suggested that an environmental assessment and report could prompt discussions with peers illuminating how different on-farm practices contributed to assessment results:

- *The other thing that’s interesting about comparing reports like he’s doing is if I compare my report to you, and we’re different. I can say, “Well, what are you doing?” … We’re all dairy farmers, and we can say, “Well, yours is different than mine. Why do you think that is? What practices are you doing that makes them different?” [MN1]*
- *I think it builds trust in the system because it encourages them to see that everyone’s getting a fair shake, but also what everyone else is doing. And I think that’s a big piece for me if you want it. You have to encourage farmers to talk to each other about it in a positive way. They can ask the experts all they want, … they’re going to check in with each other and that’s who they trust. [MN2]*

In addition to prompting discussions with peers, participants commented that an environmental assessment could start conversations with other stakeholders.

- *Yeah, I think if you show this to your veterinarian, you’re going to have a bunch of questions. If you show this to food scientists it’s going to be another one. And everybody wants their own details. And I think to producers, they are going to have other set of questions, which I think they’re all valid. [SD2]*

Still, participants stated that the environmental assessment process needed to inform and support continued improvement. For example, farmers and advisors offered that environmental assessments should provide information on leverage points for influencing environmental performance.

- *I would like to see too what are the things that we know do help you … and to what degree. We can’t always do everything, but what are the ones that help you make the most impact? There must be some. I know this stuff is refining and improving all the time. I look- [SD2]*
- *Yeah, it’s really easy to read. But as a scientist, it’d just be nice to know, can you even move the bar? If your standard deviation is this big, there’s not a lot you’re going to do about that. [MN1]*
- *Be interesting to see on one of these reports too, if we had a best practice dairy farm, like if we made up a dairy farm that was best practice, what would this report look like? [MN1]*

These statements illustrate that participants valued aspects of environmental stewardship assessments that informed on-farm decision-making. Sustainability assessment programs are designed to elicit a response, i.e., a change in farm management practices (Espeland and Sauder, 2007). However, environmental stewardship assessments vary in their effectiveness at supporting on-farm decisions (Coteur et al., 2020). Meul et al. (2014) found that lifecycle assessments could serve a role as decision support tools, although they suggested that the complexity and focus of decision support tools should depend on whether their intended users are farmers or advisors. As Arulnathan et al. (2020) emphasized, the impact categories represented by an ES assessment heavily influence its usefulness as a decision support tool. Critically, the FARM ES Version 2 assesses a narrow set of environmental impacts in relation to productive outputs and excludes social and economic factors. To enable the use of FARM ES Version 2 in supporting on-farm decisions, further developments could improve the model’s description of environmental, economic, and social processes involved in ES.

### Feasible Areas of Improvement

Improvement strategies deemed by participants to be accessible and profitable (or at least resulting in no financial losses) are shown in Table 3. Accessible and profitable strategies generally differed across regions. However, certain strategies were rated as accessible and profitable by two or more regions, indicating that these strategies may be feasible for many farms in the Upper Midwest. Importantly, accessible and profitable strategies reflect both the material and structural characteristics of regional livestock systems and participant’s individual beliefs. Participant judgements of accessibility and profitability likely depend on their personal knowledge and experiences related to each improvement strategy, which were likely more developed for well-established or popular improvement strategies. Our results do not span the entire spectrum of possible innovations to environmental management of dairy farms, rather, they reflect the management strategies deemed currently feasible by farmers and advisors.

**Table 3.** Count of regions rating each improvement strategy as accessible and profitable (or at least not resulting in financial losses)^1^

Main category^2^	Improvement strategy	Count
Feed Production	Cover cropping	4
	No-/Minimum-tillage Precision nutrient management	2
	Direct injection of manure Microbial products Nutrient management Precision ag in crop production Seed treatments	1
Animal Production	Genetic improvement	3
	Feed additives	
	Herd/facility management technologies	2
	Altering feeding behavior Cow comfort & well-being Diet/herd management Extended lactation	1
Manure Management	Anaerobic digesters Composting manure Manure aeration	1
Energy	Fossil fuel to electric motors	2
	Facility design Geothermal energy Precision ag in milk harvesting Robotics Solar energy Ventilation technologies	1

^1^Regions included Central Minnesota (MN1), Southeast Minnesota (MN2), East-central South Dakota (SD1), Southeast South Dakota (SD2), and Northeast Nebraska (NE1).

^2^Improvement strategies are categorized based on their expected impacts on the greenhouse gas footprint.

Participants selected cover cropping, no-till and minimum tillage, and precision nutrient management as feasible improvement strategies related to feed production. Focus groups reported that cover cropping was moderately accessible. Across regions, ratings of cover crop profitability varied from neutral to very profitable. For the region where cover cropping was viewed as very profitable, participants mentioned that local government mechanisms incentivized cover crop adoption above state and federal mechanisms. No-till and minimal tillage was rated as financially neutral (n = 2 regions) and neutral to moderate in terms of accessibility. In discussions, several participants clarified that minimum tillage was more feasible than no tillage, which greatly limits manure application options. Precision nutrient management was considered moderately profitable and accessible (n = 1 region). Participants identified some strategies as priorities for further development that they considered currently infeasible. This included irrigation technologies, changes to commodity sourcing, changes to equipment used in crop production, and expansions in available feed sources.

With respect to reducing environmental impacts directly associated with animal production, our participants proposed genetic improvement, herd and facility management technologies, and the feed additive monensin as feasible means of improvement. Focus groups generally viewed genetic improvement as very accessible and very profitable. Participants primarily discussed the use of herd and facility management technologies to enhance productivity, diluting environmental impacts when unitized over a greater output of milk.

Across regions, focus groups reached less consensus surrounding feasible improvement strategies related to manure treatment. Technologies such as anaerobic digestion, composting, and aeration were rated as accessible within certain areas. However, these strategies were ruled out as infeasible in most regional focus groups. Several regions (n = 3) suggested that technologies to recover nutrients and water from manure were a priority for further development but were currently not profitable. Likewise, covered manure storage was considered accessible but unprofitable (n = 2 regions).

When considering greenhouse gas impacts associated with on-farm energy use, two regions indicated that converting from fossil fuel usage to electric motors was accessible and profitable. Focus group participants mentioned that energy-saving measures and technologies were already employed to a large extent by dairy farms in the Upper Midwest. Some participants suggested that additional improvements in energy efficiency would require further technological innovation rather than greater implementation of existing technologies. In general, focus groups suggested that converting from fossil fuel usage to electric motors was accessible, although they differed in ratings of profitability. One focus group suggested hydrogen power as a priority for further development, although they rated it as currently infeasible.

### Shared Responsibilities in Enacting Progress

**Figure 2.**
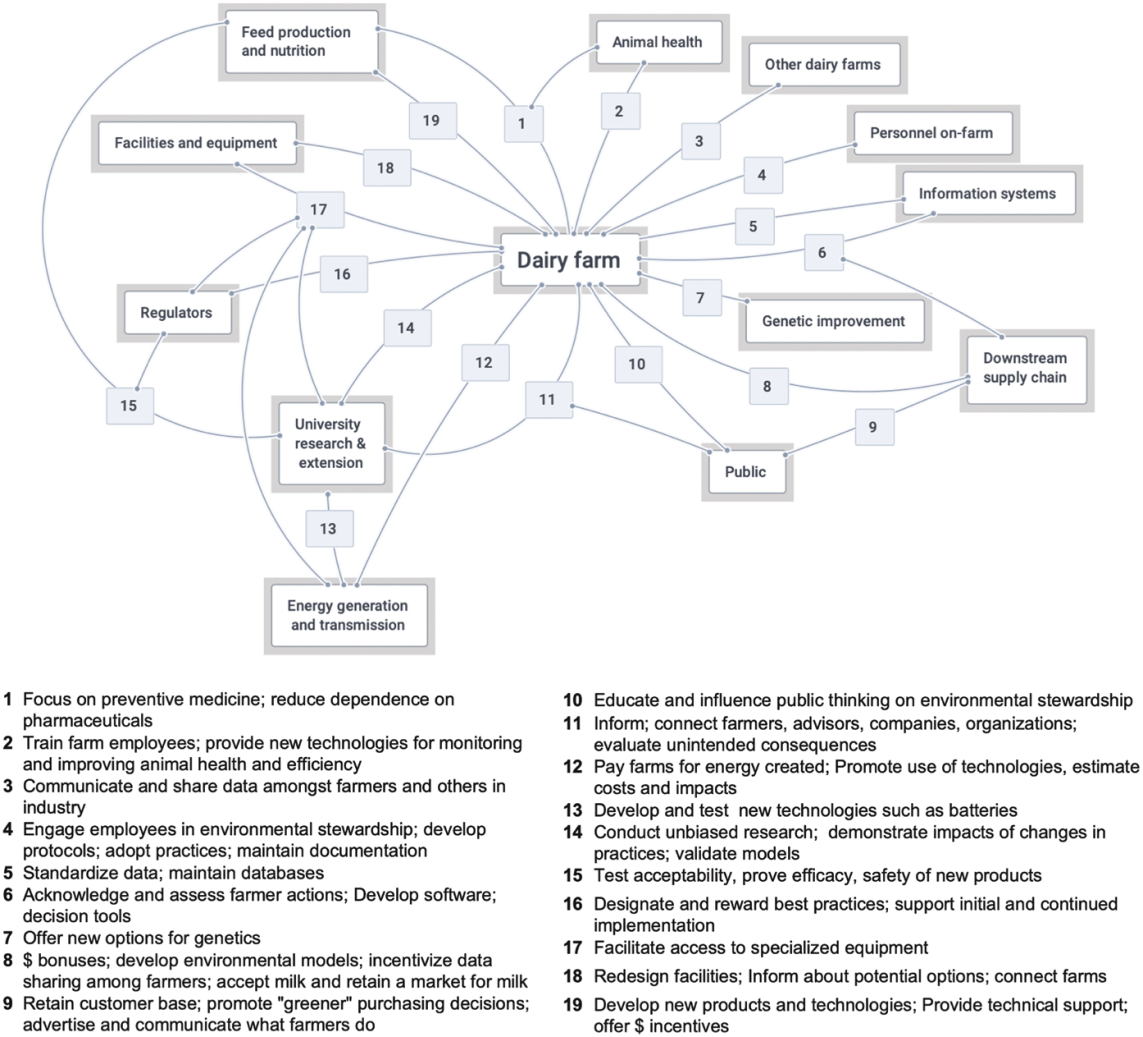
Summary of key actors and actions involved in implementing environmental stewardship in the dairy sector according to farmer and advisor participants in focus groups in 5 regions in the U.S. Upper Midwest.

Figure 2 shows a map of key actors and actions involved in dairy industry environmental stewardship, summarized from farmer and advisor focus groups. Participants described how ES progress required access to new equipment and technologies, changes to financial incentives, and restructuring of information and communication systems in the dairy sector. In general, farmers and advisors communicated that dairy farms were central to advancing environmental stewardship. However, many actions relied on collaboration across the dairy supply chain, supporting industries, and regulatory bodies.

Participants recommended numerous ways to restructure socio-material conditions in the dairy sector. Farmers and advisors mentioned that many environmental stewardship improvements required access to specialized equipment. For example, several participants commented that changes in cropping practices such as injecting manure or planting cover crops necessitated that farms purchase, rent, or borrow equipment, or hire a contractor. Farmers and advisors offered that grants and equipment sharing and rental programs via state and local government, non-governmental organizations, universities, and companies assist dairy farmers with implementing capital-intensive environmental stewardship practices. Research has shown gaps between the intended and actual implementation of climate change mitigating practices (Niles et al., 2016), which suggests that farmers may have the motivation but not the means to implement environmental stewardship practices.

According to focus groups, improving environmental stewardship required innovations in technologies and practices. Participants voiced that supporting industries, universities, and regulatory bodies could work together in developing and testing new products and technologies aimed at enhancing productive efficiency and reducing environmental impacts. Importantly, participants emphasized a need not only for innovation, but support during initial and continued implementation. To support implementation, participants mentioned that farms need guidance on how to integrate new products and technologies into their operations. As emphasized by Ritter et al. (2020), on-farm changes often require investments and constitute financial and operational risks. Similar to Thompson et al. (2021), our participants expressed needs not only to support the initial adoption of environmental stewardship practices but also to increase the extent and duration of their use. As suggested by Hoes and Aramyan (2022), policies and programs aimed at improving environmental stewardship need not only to destabilize unsustainable practices but also to support “pioneering farmers” in implementing innovative practices.

Participants also mentioned restructuring financial incentives across the dairy supply chain. For instance, farmers and advisors discussed how companies could offer financial incentives to reduce the costs incurred by dairy farms to improve environmental stewardship. Likewise, participants stated that energy and electric companies and regulators could enhance financial rewards for farmers generating renewable bioenergy. Participants emphasized that the financial incentives associated with synthetic fertilizer sales hindered the use of manure as a renewable fertilizer and could be restructured. When considering processors, retailers, and consumers, participants mentioned that purchasing decisions and prices should financially reward farmer involvement in environmental stewardship. For example, participants suggested that processors and cooperatives could offer financial bonuses to recognize excellence and improvement in ES. Research on milk quality improvement showed individual differences in the responses of Dutch dairy farmers to extrinsic motivators such as financial penalties and incentives (Valeeva et al., 2007). Through interviews with English dairy producers, Coyne et al. (2021) found that financial incentives were a major motivator to join agri-environmental schemes, although a minority of their participants indicated that financial incentives had directly induced changes in their behavior. Therefore, more research is needed to understand extrinsic factors that effectively motivate environmental stewardship improvement.

In addition to altering the dairy industry’s financial structure, participants proposed a need to reorganize information systems related to ES. For example, dairy associations, processors, and information technology industries were mentioned as key players in standardizing data collection, sharing, and archival. Farmers and advisors positioned processors and cooperatives as key intermediaries involved in developing and implementing environmental stewardship assessments and communicating results with the public. Moreover, participants suggested that processors could work with software developers to design environmental stewardship decision-support tools. As agricultural data has become richer and more free-flowing, farmers and advisors participate in increasingly complex information-sharing and decision-making systems (Brown et al., 2023). Cabrera and Fadul-Pacheco (2021) reviewed developments in dairy data systems, suggesting that integration of multiple data sources can contribute to managing environmental impacts from farm systems. Other authors pointed out a specific need to integrate environmental stewardship assessments with the data systems used by advisors (de Mey et al., 2011). Therefore, collating multiple data sources has the potential to characterize on-farm processes in greater spatial and temporal resolution, potentially offering new means to manage environmental impacts.

In addition to data management systems, participants drew attention to information sharing through social structures. For example, participants suggested that university extension could connect networks of farmers, advisors, companies, and organizations involved in environmental stewardship to facilitate progress. Focus groups further discussed how communication within and amongst farm personnel could inform and motivate environmental stewardship improvement. For example, promoting employee engagement in ES, highlighting exemplary farms, and facilitating information-sharing amongst farmers were mentioned as key actions. Participants mentioned that effective protocols and training had drastic implications on environmental performance, in part through effects on herd health and efficiency. Nguyen et al. (2019) emphasized a need for shared learning spaces enabling agriculturalists to co-construct procedural knowledge related to ES. In other words, open communication can advance collective knowledge of *how to* improve ES. Through focus groups with Canadian dairy farmers, Ritter et al. (2020) described a similar need for farmers to learn from each other to improve animal welfare outcomes. Importantly, these participants clarified that motivating change required not only informal peer-to-peer interactions but also industry-level goals and regulations.

## RECOMMENDATIONS

Environmental assessments such as the FARM ES Version 2 can enable voluntary engagement of dairy sector stakeholders in ES. Because the dairy farm is a locus for decision-making, improving farmer and advisor buy-in to assessment processes is critical to reducing the cradle-to-farmgate environmental impacts of milk production. Our findings suggest several areas for future work:

1) **Improving accuracy, spatial and temporal resolution, and flexibility of environmental stewardship assessments to increase their value to farmers and advisors**. Our findings revealed that assessments such as FARM ES are valued by stakeholder groups (e.g., farmers, advisors, consumers, processors) for different reasons. Whereas our participants considered the FARM ES Version 2 as valuable for processors and retailers, they suggested its value to farmers and advisors could be improved. Specifically, the FARM ES assessment could be made more customizable to particular farms and geographic regions and more interoperable with data systems used by advisors. Additionally, the model can be made more transparent in describing calculation methods and benefits to various stakeholders.
2) **Promoting action through iterative assessment processes and post-assessment social and technical support.** Our results showed that the FARM ES Version 2 could serve as a starting point to estimate environmental performance at one point in time. However, participants suggested that interactions with peers and technical experts following an assessment could motivate and inform action on assessment results. Further, participants expressed a desire to utilize initial assessments as a baseline for continued improvement. This suggests a need to consider the assessment’s responsiveness to management changes and to adapt assessment procedures for when the assessment is used repeatedly.
3) **Building human capacity for ES by strengthening networks of people, companies, and organizations involved in specific ES challenges or within specific regions.** Our findings showed that several ES improvement strategies were perceived as currently feasible but not widely adopted. This suggests it is possible to motivate a greater extent of implementation by altering social conditions.
4) **Developing new technologies and increasing access to equipment and resources required for ES improvement**. Our participants expressed a need for developing and proving tools to manage ES, particularly in certain areas such as feed production, manure management, and farm information systems.

## CONCLUSIONS

Farmer and advisor participants reported that the FARM ES Version 2 was an accessible assessment that provided basic regional comparisons and starting points for implementing management changes with peer and advisor support. Conversely, some participants expressed concerns surrounding the opacity of model calculations and the accuracy of resulting outputs. Farmers and advisors expressed a need to ensure that assessment processes were inclusive, fair, and beneficial to various dairy industry stakeholders beyond their clear benefits to processors and retailers. Across five regions, participants identified several improvement strategies that they deemed currently feasible for dairy farms in the Upper Midwest. Feasible strategies that primarily addressed feed production impacts included cover cropping, conservation tillage, and precision nutrient management. Participants suggested genetic improvement, herd and facility management technologies, and the conversion of fossil fuel to electric motors as means of reducing environmental impacts associated with the animal herd and facilities. Finally, participants mapped the responsibilities of farmers, supporting industries, regulators, and universities in restructuring socio-material conditions in the dairy sector to improve environmental sustainability. Our findings illustrate the challenge of designing accurate and useful environmental assessments that serve the needs of multiple stakeholders and effectively motivate actions.

## Supplementary Material

txaf038_suppl_Supplementary_Materials
