# Supplementary material for: Building value for dairy farmers and advisors in the Farmers Assuring Responsible Management Environmental Stewardship Program
Source: Transl Anim Sci. 2025 Mar 25;9:txaf038. doi: 10.1093/tas/txaf038 (PMC12035815; doi:10.1093/tas/txaf038)

SUPPLEMENT 1

Facilitator Process Guide
Building Value in Baselines
Topic 1: The power of baseline metrics
2023

I. Setting the Stage (20 minutes) 3
- a. Welcome Remarks 3
- b. Ice breaker 3
- c. Guideposts 3

II. Discuss Issue Snapshot and Aspects of the Problem (10 minutes) 5

III. What does a FARM ES report look like? (20-25 minutes) 5
- a. Overview 5
- b. Inputs 6
- c. Outputs 6

IV. What is the value in FARM ES reports? (20-30 minutes) 7

V. Reflection and Action Prioritization (10 minutes) 7

VI. Closing (5 minutes) 8

I. Setting the Stage (20 minutes)

a. Welcome Remarks

Host: Thank you all for coming.

[Introduce yourself]

Today we will all be taking part in an open discussion about environmental sustainability assessments. We are not coming to any specific solutions, and each of us may take away our own conclusions. We hope everyone is willing to listen carefully and build on the perspectives of others.

I/We are here to serve as *facilitator*. I/We intend to be a neutral discussion leader for the conversation.

[Distribute photo / video release form]

We are recording audio for purposes of capturing ideas that we know we will not be fast enough to write down in the moment, not who said what. We are interested in themes that arise from this discussion, as well as similar conversations across the region. We can use each other's names throughout this conversation, because we will delete all names when we transcribe the audio to text. The audio file will also be deleted to protect your privacy.

The photo release allows us to document the active involvement of dairy farmers and industry representatives in working toward industry sustainability goals. We want to share a few photos in presentations about this work to academic audiences and the public.

b. Ice breaker

To begin our conversation, can everyone please introduce yourself to everyone else at the table by sharing your name and your favorite flavor of ice cream?

c. Guideposts

Before we get started we'd like to suggest some ways we can ensure a fair, responsive and productive conversation. Can we hold each other accountable to the following guideposts?

1. Listen carefully and honor the value of each other's contributions.
2. Avoid dominating the conversation or talking over one another.
3. Ask clarification questions and build on what others have said.
4. Allow for opinions to change throughout the discussion.
5. Feel free to jump into the conversation, or show your hand to indicate you want to add something.

[Ask participants and write on whiteboard or large paper]

- Does anyone have other ideas for how to keep our discussion productive today?

[Order meal?]

II. Discuss Issue Snapshot and Aspects of the Problem (10 minutes)

Host: Over the last year, I/Erin was part of conversations and discussions about the net zero initiative and environmental stewardship through various dairy-centric meetings in the Midwest. From those conversations we learned that approximately 45% of participants (farmers and dairy farm advisors) are interested in figuring out their baseline for sustainability metrics like carbon footprints.

[Ask participants]

- Could anyone volunteer some ideas…what comes to mind when you think of the term baseline?

We think of baselines as a starting point. We are not solving any problems today. Instead, we will focus on the value of baselines and assessments, now, or in the future. In later conversations, we will work together on how Midwest farms can act on these results.

The FARM ES program took years to create and involves fairly complicated calculations. We did not create the program and we do not control how it works. Instead, we are also users of the reports, just like you. To keep the conversation focused, we are going to focus on how the reports are useful, not how the results are calculated.

III. What does a FARM ES report look like? (20-25 minutes)

a. Overview

Host: Our first task today is to figure out what is included in a FARM ES report. We are going to look at some "baseline" assessments of carbon and energy footprints for example farms today generated using the same program as the FARM Environmental Stewardship (ES) assessments. These sheets are not specific to any of your farms – they are fictional. However, they are set in this state and based on farm data. We will start with just one farm.

[Distribute printed Test Farm 1 to participants.
Make sure everyone has a pen or pencil]

b. Inputs

Host: You should have a five page report. Feel free to mark it up to make notes. First, let's take a few minutes to look through the first three pages of the report. These pages show the farm records used in the calculations. Sometimes we refer to these as the "inputs" used in the calculations.

[Offer 1-2 minutes to look through p. 1-3]

[Ask participants about INPUTS - show of hands or open discussion]

- How many of you have seen one of these before? Something similar?
- What are your impressions?
- What initial questions do you have?
- Does anyone have any questions about specific sections in pages 1-3?.
    - Details about the farm, evaluator (p. 1)
    - Milk and beef production averages (p. 1)
    - Herd profile (p. 1)
    - Energy types and sources (p. 2)
    - Feed rations (p. 2)
    - Self-produced Crop Percentages (p. 2)
    - Manure Management Systems (p. 3)
    - Nutrient Management Plan (p. 3)

c. <u>Outputs</u>

Host: Next we will turn our attention to pages 4-5, which show the "outputs" calculated by the FARM ES program. This tells us the farm's performance on several Environmental Stewardship metrics. There is a lot to discuss, but let's start by sharing some initial reactions.

[Ask participants about OUTPUTS - show of hands or open discussion]

- What are your overall impressions of the figures and tables here?
- What initial questions do you have?
- Let's look at this section by section. There are three sets of values - one for the individual farm, a regional average, and a national average.
    - Your Farm Greenhouse Gas Emissions (p. 4)
    - Your Farm Energy Use (p. 4)
- Finally, what do you notice about the final table?
    - Gas Type Breakdown (p. 5)

IV. <u>What is the value in FARM ES reports? (20-30 minutes)</u>

Host: Now that we know how to interpret the report, next we're going to look at another farm. "Test Farm 1" was a small herd with 150 lactating cows. "Test Farm 2" is a larger herd with 1500 lactating cows. At this stage, we want to hear a lot more from you about your impressions of the results.

Let's take a few minutes to look at another farm.

[Distribute Test Farm 2 report]

[Ask participants to volunteer their impressions, or go around the table one-by-one]

- What can you appreciate about these assessments?
- What value is there in seeing multiple reports?

- What changes would add value to this baseline report?
- What context is missing?

- What value does an assessment provide - now or in the future?
- This type of assessment is usually between a processor and a farm. Who else could benefit from seeing this report?

V. Reflection and Action Prioritization (10 minutes)

Host: We need to wrap up in the next 20 minutes, so let's take a moment to reflect. We gave you these assessments with very little time to digest them.

- Did any comments shared today spark additional thoughts you want to build on?
- A set of baseline numbers are in themselves, part of environmental stewardship - understanding one's impact on the environment. Is this type of assessment enough to spur discussion about actions to change any of these values? What else is needed?
- How can a farm and advisors use this assessment to ideate?
- How do you see FARM ES reports contributing to your own farm (or your work with client farms)?
- What obstacles do you see in implementing this in practice? Do you have suggestions for overcoming these challenges?

Today's focus was the assessment reports - as a first step that farms or people can take in their sustainability journey. We hope you are willing to re-engage with us over another dinner in the next month to see how we move from understanding, to action.

You can take the reports with you and if more questions arise, we welcome those – preferably at our next focus group meeting. To help us plan, what feedback or questions would you like to discuss at our next meeting? You can share them on a piece of paper or sticky note anonymously.

[Distribute sticky notes or papers to participants]

VI. Closing (5 minutes)

Host: Thank you everyone for coming to the focus group and sharing your experiences.

As a reminder, we will delete all the audio files and anonymize these conversations to protect your privacy.

SUPPLEMENT 2

Facilitator Process Guide
Building Value in Baselines
Topic 2: Moving the needle – what makes sense for our area?
2023

I. Welcome and Recap (5 minutes) 3
- a. Welcome Remarks 3
- b. Ice breaker 3
- c. Guideposts 3

II. Follow-up from First Meeting (15-20 minutes) 3
- a. Recap 3
- b. Additional thoughts 4

III. Brainstorm Management Changes (25-30 minutes) 4
- a. Individuals free-write 4
- b. Group expands lists for each category 5
- c. Group narrows down to potential options for the region (e.g., for Southeast MN) 6

IV. Map priorities (5-10 minutes) 6

V. Expand on further development priorities (exciting long-shots) (10-15 mins.) 7

VI. Reflection and closing (5-10 minutes) 8

I. Welcome and Recap (5 minutes)

a. Welcome Remarks

Host: Thank you all for coming.

[Introduce yourself]

[Distribute photo / video release form for people who missed it]

As a reminder, we are recording audio for purposes of capturing ideas that we know we will not be fast enough to write down in the moment, not who said what. We can use each other's names throughout this conversation, because we will delete all names when we transcribe the audio to text. The audio file will also be deleted to protect your privacy.

As a reminder, the photo release allows us to document the active involvement of dairy farmers and industry representatives in working toward industry sustainability goals. We want to share a few photos in presentations about this work to academic audiences and the public.

b. Ice breaker

Host: To refresh everyone's memory, can each of us please introduce ourselves by sharing your name and your favorite type of cheese?

c. Guideposts

Host: Last time, we established some guideposts to promote a fair, respectful, and productive conversation. The guideposts are posted here.

[Share guideposts on prepared sheet via easel]

II. Follow-up from First Meeting (15-20 minutes)

a. Recap

Host: Any follow-up thoughts from the first meeting? Is there something you wished you said, or did any new thoughts come to you on the drive home?

Your comments in the first meeting a month ago were invaluable. Three farm-focused groups, including this one, met in the last month (more to come). Common themes that arose from those conversations were:

- The FARM ES assessment is simple and easy to complete for most farms.
- There are ways the FARM ES reports can be improved to make them more useful for decision-making.
- Accuracy, consistency, fairness, and privacy are important considerations.

We also had a meeting with processor representatives. Some of the main findings from processors were:

- Their customers (retailers) are requesting aggregated environmental impact data.
- They are also learning how best to interpret and use the FARM ES reports.
- They often get questions from farmers about how environmental data will be used.

Since we are in the midst of these focus groups, these summaries may slightly change. But we have shared these high-level themes back to Midwest Dairy, and are arranging a sit-down with US Dairy to make sure your voices are heard.

b. <u>Additional thoughts</u>

[Ask participants]

- Does this surprise you?

- As a focus group participant, what makes you feel your input is valued?

III. <u>Brainstorm Management Changes (25-30 minutes)</u>

a. <u>Individuals free-write</u>

Host: As we move into this next phase, we want to share some basic assumptions as starting points for this conversation:

- The majority of farmers and farms are doing their best.
- Environmental decisions are guided by more than one number on an assessment, including economic and operational factors.
- Continual improvement starts with knowledge.
- There is power in the masses in the adoption of practices or technologies.

At this meeting, we want to move forward from baselines and collectively think through ways to potentially change GHG metrics. GHG emissions are only one aspect of environmental impact, so we welcome other environmental stewardship measures, and we can discuss if and how they may relate back to GHG metrics. **We are not worried so much about the degree of change in the metric, but rather the opportunity for change or adoption.**

Let’s take a moment to look through some management changes suggested by US Dairy. Similar to the FARM ES reports, this figure groups farm impacts into four categories: enteric methane, energy, feed, and manure management.

[Give participants a handout with USDairy figure and open table for additions]

Enteric methane	Energy	Feed production	Manure Mgmt.

- Take a moment to write down - What would you add to these lists? If you have expertise in a particular category, please feel free to focus there. We want to be creative and generate as many options as possible!

b. Group expands lists for each category

Host: Let's look one category at a time.

[Show on easel something similar to below, 1 page per category]

Enteric methane<br>Diet management<br>Genetic improvement<br>Herd management<br>Cow comfort & well-being<br>Feed additives

- First, what do each of these USDairy suggested changes mean to you? For example, how do you define diet management?
- What strategies did you think of to add to this category?

  [Facilitator lists participant-generated strategies in white space below, repeating the process for each of the four categories.]

c. Group narrows down to potential options for the region (e.g., for Southeast MN)

Host: Next we are going to think more practically, and focus on this region. Think of the dairy farms you are familiar with, approximately within a 50 mile radius.

- **Implementation priorities**: Which of these options are already widely- and intensively-used or can readily be implemented for most dairy farms (>50% of dairy farms) in this

region, within the next 5 years? Why?

- **Further development priorities**: Which of these options are close to being implementable, but something needs to change to improve on-farm adoption in this region? Why?

- **Not currently a priority**: Which of these options are highly unlikely to be useful to farms in this region? Why?

  [Facilitator crosses off unrealistic, circles items deemed already done (by >50% of farms), adds * to realistic items; facilitator or assistant picks priority items to write on sticky notes]

IV. Map priorities (5-10 minutes)

Host: [Discuss immediate implementation priorities, if any. Is there opportunity for change, or is this already done by >50%]

Going back to our starting point, remember **we are not worried so much about the degree of change in the metric, but rather the opportunity for change or adoption.** Starting with some of the best options we identified in the middle (further development category), we are going to use a graph to get even more detailed. On the x axis, we have how profitable/unprofitable the strategy is to implement. In other words, how much will it cost (negative) or pay off (positive) for a farm to adopt this strategy? On the y axis, we have how easily accessible the strategy is for farmers. Is this strategy difficult to enact, or accessible to everyone?

[Show something similar on the easel]

Unprofitable	Available/accessible<br>Profitable
Unavailable/inaccessible	

- Where should we place the sticky note for ____[Diet management]____? Why?
- Does anyone disagree? Why?

[Facilitator repeats this for each sticky note]

- How does the map look? Are there details or contingencies missing?

[Facilitator lists some details next to sticky notes]

Looking at the accessible/profitable corner of the map, we have identified the most realistic management strategies that would work on many farms in this region within the next five years.

- Are there any obstacles that prevent these strategies from being immediately used on farms?
- For which situations do you expect implementation will be easier, or more challenging?

V. Expand on further development priorities (exciting long-shots) (10-15 mins.)

Host: We have been very realistic. Next, let's be more imaginative and return to the further development priorities we identified. For today, we will not discuss any unrealistic options.

[Facilitator turns back pages on easel to previous]

- Which of these management strategies is exciting to you, and why?
- What could make this management strategy feasible?

VI. Reflection and closing (5-10 minutes)

Host: We discussed a complex topic today where many people feel confused about the next steps to take. To finish up, we want to challenge you to think about how this applies to your own farm or your work in advising farms.

- How does this relate to you? What impressions or actions will you take away from this meeting?

In our next meeting, we will find ways that we can work together across the dairy industry to promote action. The action steps will look different for farms with different characteristics. Advisors will play a different role than farmers.

- Are there specific topics or ideas you would like to explore when it comes to prioritizing action steps?

Thank you everyone for coming to the focus group and sharing your experiences. As a reminder, we will delete all the audio files and anonymize these conversations to protect your privacy.

- Does ____ date and time work for our next meeting?

SUPPLEMENT 3

Facilitator Process Guide
Building Value in Baselines
Topic 3: Shared responsibility – what is the basic step for the promotion of action?
2023

I. Welcome and Recap (20 minutes) 3
- a. Welcome Remarks 3
- b. Ice breaker 3
- c. Guideposts 3

II. Follow-up from Prior Meetings (10 minutes) 3
III. Overview of today's meeting 4
IV. Selecting focus strategies (5-7 minutes) 4
V. Shared responsibilities (20-30 minutes) 4
- a. Local goals 4
- b. Industry goals 5

VI. Building momentum, e.g., peer groups 6
VII. Closing and Evaluation (5 minutes) 7

I. Welcome and Recap (20 minutes)

a. Welcome Remarks

Host: Thank you all for coming.

[Introduce yourself]

[Distribute photo / video release form for people who missed it]

As you may know by now, we are recording audio for purposes of capturing ideas that we know we will not be fast enough to write down in the moment, not who said what. We are interested in themes that arise from this discussion, as well as similar conversations across the region. We can use each other's names throughout this conversation, because we will delete all names when we transcribe the audio to text. The audio file will also be deleted to protect your privacy.

As a reminder, the photo release allows us to document the active involvement of dairy farmers and industry representatives in working toward industry sustainability goals. We want to share a few photos in presentations about this work to academic audiences and the public.

b. Ice breaker

To refresh everyone's memory [and for any new attendees], can each of us please introduce ourselves by sharing your name and a hobby you enjoy outside of dairying?

c. Guideposts

Last time, we established some guideposts to ensure we could hold a fair, respectful, and productive conversation. The guideposts are posted here.

II. Follow-up from Prior Meetings (10 minutes)

Host: It's been almost two months since our first meeting where we focused on FARM ES reports. In our second meeting, we brainstormed ideas for "moving the needle" on environmental stewardship performance. We were able to identify a set of strategies for improving farm environmental footprint that were both profitable and immediately accessible to farmers.

[Ask participants]

- Does anyone have thoughts or reflections on the most recent meeting?

We also met with representatives of Midwest Dairy and Dairy Management, Inc. (DMI) to share some of your ideas. What we learned:

- Midwest Dairy and DMI are particularly interested in hearing your responses for the second and third meetings.

- They expressed a desire to learn more about how to support the state's dairy industry in enacting farm environmental stewardship, e.g., through producer-led efforts.
- Additionally, they are planning improvements in the next version of FARM ES program that your feedback can inform.

Our teams from SDSU, University of Minnesota, and University of NE are working to serve as a bridge between farmers, advisors, and dairy industry organizations. At the end of the year, we will conclude this series of focus groups. However, there is potential to continue this work.

[Ask participants]

- What would you like to see from these collaborative efforts in the future, after this series of focus groups concludes?

III. Overview of today's meeting

Host: At today's meeting, we want to return to a very practical focus. The dairy industry comprises many actors at different levels, with different priorities. Our team views environmental stewardship as a shared responsibility of many different players in the dairy industry. That said, we assume that many people will have to take action in order for the dairy industry to collectively make progress on environmental goals.

IV. Selecting focus strategies (5-7 minutes)

Host: For this group, we generated a list of management strategies that were both accessible and profitable ways for producers in this region to improve environmental performance:

- Facility design
- Cover crops
- Genetic improvement and reproduction strategies
- Herd health
- Cow comfort and herd management
- Manure as a renewable fertilizer
- Geothermal energy
- Seed treatments

To get started, we would like to pick three from this list that our discussion will focus on tonight. We can use a show of hands to vote quickly. [Vote for each item on the list, writing # of hands up on the easel, to select top three].

V. Shared responsibilities (20-30 minutes)

Host: Now, for each of these strategies, we want to think together about our responsibilities in making progress. We generated a list of who might be "key players" in environmental stewardship in the dairy industry, which is open to additions/revisions.

- ❖ Technical support
- ❖ Repro companies

- ❖ Feed industry
- ❖ Nutritionist
- ❖ Veterinary services
- ❖ Farm Bureau
- ❖ Electrical cooperative/Energy companies
- ❖ NRCS
- ❖ Agronomists
- ❖ Banker/financial consultant
- ❖ Extension
- ❖ Farmers
- ❖ Midwest Dairy
- ❖ Processors
- ❖ Universities
- ❖ Government organizations
- ❖ Other industry organizations
- ❖ Other players

← [For each of the three selected focus strategies (e.g., cover crops), repeat the section below for next levels] →

- Who would you identify as the key players in promoting progress in adoption and advancement of [this focus strategy, e.g., cover crops]?
- Who can play a supporting role?

[Facilitator lists roles on the easel. If participants do not spontaneously share, the facilitator asks participants to elaborate on the actions involved in this role]

[Ask participants, Repeat for each desired outcome.]

- How can we work backwards to achieve this goal? Fill in the middle column with actions.

**If this key actor**	**Does/keeps doing these actions**	**Then we will achieve**
Farmers		Most (>80%) of farms can adopt this practice.<br><br>[What other outcomes? Participants can generate implications]
Advisors		
Processors		
…		
Midwest Dairy		

Most (>80%) of farms can adopt [this practice]

<table>
<tr><th>If this key actor</th><th>Does/keeps doing these actions</th><th>Other outcomes (positive negative)</th></tr>
<tr><td>Farmers</td><td></td><td rowspan="5">[What other outcomes? Participants can generate]</td></tr>
<tr><td>Advisors</td><td></td></tr>
<tr><td>Processors</td><td></td></tr>
<tr><td>…</td><td></td></tr>
<tr><td>Midwest Dairy</td><td></td></tr>
</table>

VI. Building momentum, e.g., peer groups

Host: Across MN and SD groups, we have heard it suggested that farmers and advisors would like opportunities to gather for continued discussions on farm environmental stewardship. We want to ask your ideas on how to keep building momentum for local leadership and cooperation among farmers and advisors.

WHO/WHAT

- What would make a peer group worthwhile for you to attend?
- We envisioned several possible types of peer groups. What other ideas would you suggest? WHY or WHY NOT (would you favor each approach?)
    A. similar-region peer groups - gathers people within a geographic region from various farm types. This is convenient to organize, and may offer opportunities for working together locally.
    B. similar-farm peer groups - consists of farms with similar characteristics (e.g., facility design, diet, manure storage) and similar management priorities.
    C. strategy-specific peer groups - bring together farmers and advisors interested in adopting specific new environmental sustainability practices on their farming operations (or facilitating adoption). The aim is to share experiences, facilitate troubleshooting, and stay informed about new advancements.

WHERE/WHEN/HOW

- For you individually, are there any preferences on:
    - Where to meet?
    - How frequently to meet?
- In your opinion, how should meetings be facilitated?

VII. Closing and Evaluation (5 minutes)

Host: Thank you everyone for coming to the focus group and sharing your experiences. We left about 10 extra minutes to allow for surveys. As a final request, please take a few moments to fill out our evaluation form. This will help us document our progress and factor in your feedback so we can continue to host similar events in the future.

[Distribute paper surveys to participants]

As a reminder, we will delete all the audio files and anonymize these conversations to protect your privacy.

SUPPLEMENT 4

Figure S1. Aspects of a typical dairy greenhouse gas footprint. Source: U.S. Dairy.

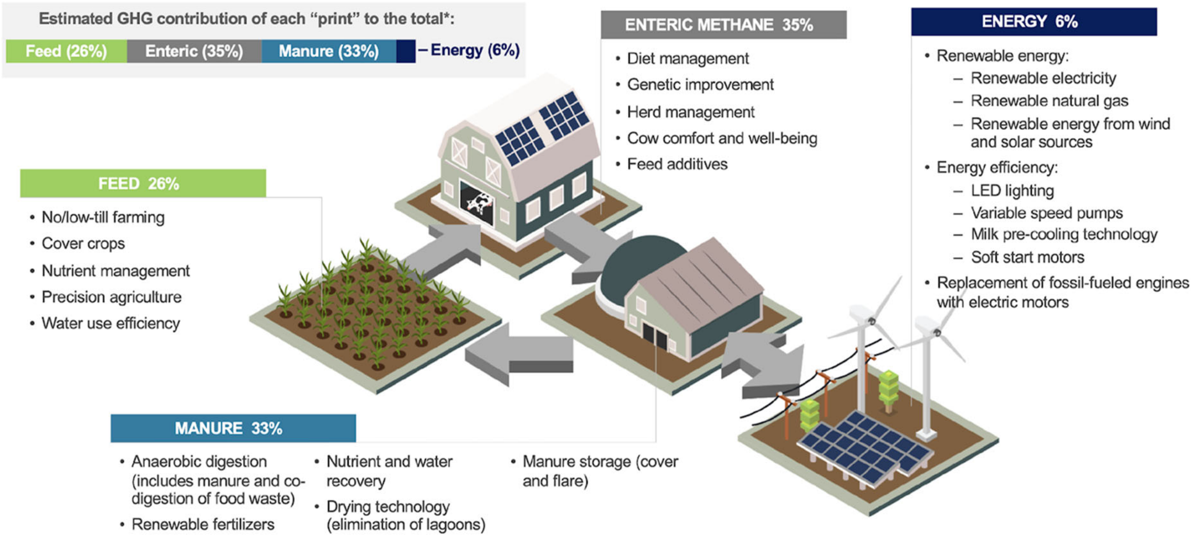

Supplement: txaf038_suppl_Supplementary_Materials [file txaf038_suppl_supplementary_materials.pdf]
